# Himalayan alpine ecohydrology: An urgent scientific concern in a changing climate

**DOI:** 10.1007/s13280-022-01792-2

**Published:** 2022-11-02

**Authors:** Ruolin Leng, Stephan Harrison, Karen Anderson

**Affiliations:** 1 Department of Geography, University of Exeter, Cornwall Campus, Penryn, TR10 9FE Cornwall UK; 2 Environment and Sustainability Institute, University of Exeter, Cornwall Campus, Penryn,, TR10 9FE Cornwall UK

**Keywords:** Climate change, Ecohydrology, Himalayan alpine zone, Remote sensing, Water–plant interactions

## Abstract

Climate change is projected to have important impacts on snow and vegetation distribution in global mountains. Despite this, the coupling of ecological shifts and hydrological processes within alpine zones has not attracted significant scientific attention. As the largest and one of the most climatically sensitive mountain systems, we argue that Himalayan alpine ecohydrological processes require urgent scientific attention because up to 1.6 billion people rely on water supplies from the mountains. We review studies from global mountain systems to highlight the importance of considering ecohydrological impacts within Himalayan alpine zones (4100–6000 m.a.s.l), explaining mechanisms for interactions between snow and dwarf plants. Our findings highlight the paucity of monitoring stations within Himalayan alpine systems. We suggest that it is likely that alpine ecological shifts will impact hydrological processes, but we found that specific mechanisms and functional relationships are missing for Himalayan systems, so the strength and direction of ecohydrological relationships is currently unknown. We advocate for more purposeful and widespread monitoring efforts below glaciers and above the treeline, calling for new experiments to query the role of small plants within the Himalayan alpine hydrological system. We outline the need for community engagement with alpine ecohydrological experiments, and we explain how new snow and vegetation products derived from remote sensing observations have the potential to improve scientific understanding of the interacting effects of warming and ecohydrological factors in this sensitive region.

## Introduction

The Intergovernmental Panel on Climate Change (IPCC) sixth assessment report (AR6) argued that the global land surface temperature is expected to increase by more than 1.5 °C in the future two decades even with current pledges under the Paris Agreement. Whilst this will be of global importance, it is known that the impacts from increasing temperatures are comparatively more intensively felt in mountains compared to other global systems (Beniston 2003; Dolezal et al. 2016).

The Himalayas represent the highest mountain systems in the world, with elevation gradient ranging from about 800 m to 8848 m above sea level (m.a.s.l), including the world’s highest peak—Mount Everest (Golovatch and Martens 2018). With their headwaters in the Himalaya, the Indus and the Ganges provide the life-supporting water for about 50 million mountain people and more than 1.4 billion people living on the plains (Ives and Messerli 1990). Accordingly, the Himalayan region is described as the ‘third pole’ and ‘Asian Water Towers’ (Bolch et al. 2012; Immerzeel et al. 2014), and the Himalaya-Ganges system is considered as one of the largest ‘highland-lowland interactive’ systems globally (Ives and Messerli 1990). Considerable scientific attention has focussed on the vulnerability, sensitivity and dynamics of glaciers, treelines or fluvial runoff with climate change in the Himalayas (Singh et al. 2012; Gaire et al. 2014; Shannon et al. 2019; Nie et al. 2021) with a goal of deepening scientific understanding of hydrological variations triggered by climate change, and possible influences on human society. Despite this extensive body of work, ecohydrological processes remain comparatively overlooked.

**Fig. 1:**
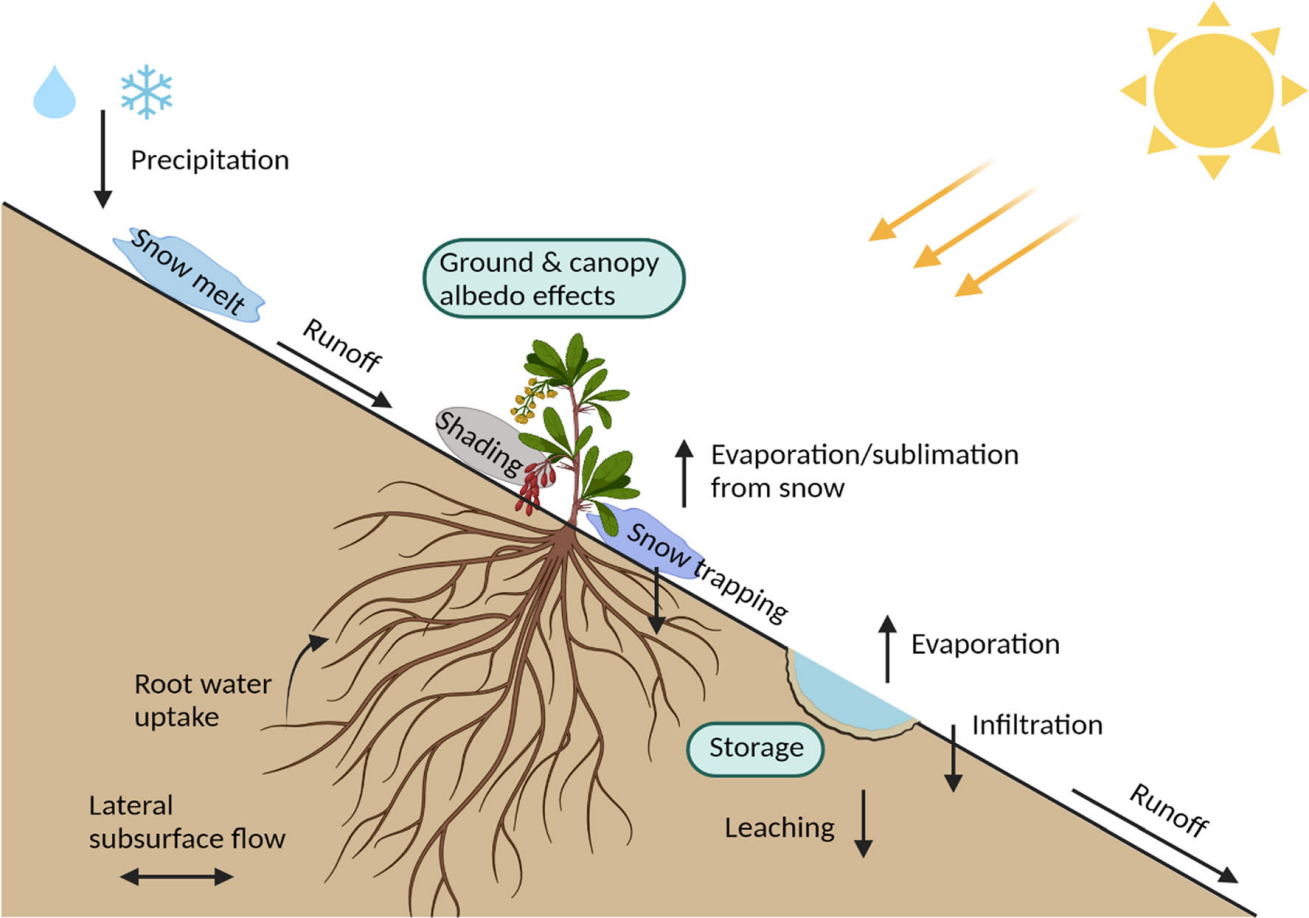
Conceptual diagram showing ecohydrological interactions in alpine systems with implications for nutrient, water and carbon cycles, and soil biogeochemistry (modified following Fatichi et al. 2016)

In the Himalaya it is crucial to consider the dominant hydrological processes contributing to runoff. In most mountain systems the majority of precipitation falls below the treeline as rain (Perry et al. 2020), whilst snow and glacier melt are dominant contributors to annual flows higher up in the alpine zone (Immerzeel et al. 2009; Lone et al. 2021). Using simulated meteorological data from the numerical Weather Research and Forecasting (WRF) model, Bonekamp et al. (2019) demonstrated that ~ 42% of the annual precipitation in the high-altitude Himalaya falls as snow. Studies in other cold climate systems such as the Arctic have shown that hydrological impacts can also arise from changes in vegetation type, density and land cover (Sturm et al. 2001; Liston et al. 2002; Naito and Cairns 2011). Thus, since snowmelt is an important contributor for subsequent overland flows, it is critical to consider water–plant interactions in alpine systems (Molina et al. 2007), since the inherent coupling between vegetation and water cycles can exert effects across a range of spatial and temporal scales (Fatichi et al. 2016; Fig. 1). Furthermore, ecohydrological functioning of water–plant interactions in the Himalayas could become more significant especially in the alpine zone, as multiple factors change with climate warming, e.g. changing albedo as snow melts and plants grow, limited ability of alpine plants to migrate or compete as temperatures warm. All these factors in combination could have a multitude of possible effects on alpine hydrological functioning (Shannon et al. 2019; Anderson et al. 2020; Körner 2021).

Following Fig. 1, we assert that a broad understanding of ecohydrological interactions in the context of Himalayan alpine systems is now needed, and whilst there has been considerable research on changing treelines and forest ecology in the region (Singh et al. 2012; Gaire et al. 2014), there remains a paucity of ecohydrological work above the treeline. A recent study in High Mountain Asia demonstrated that in the high altitude (> 4000 m.a.s.l) and non-irrigated area of the Ganges–Brahmaputra basin, the expected release from temperature limitation under future warming also highlights the importance of considering non-temperature limitations (e.g. soil characteristics, species migration, recruitment, establishment, competition and community dynamics) in mediating ecosystem responses to future climate change—evidencing the need for urgently addressing alpine plant-water relations in the Himalaya (Maina et al. 2022).

**Fig. 2:**
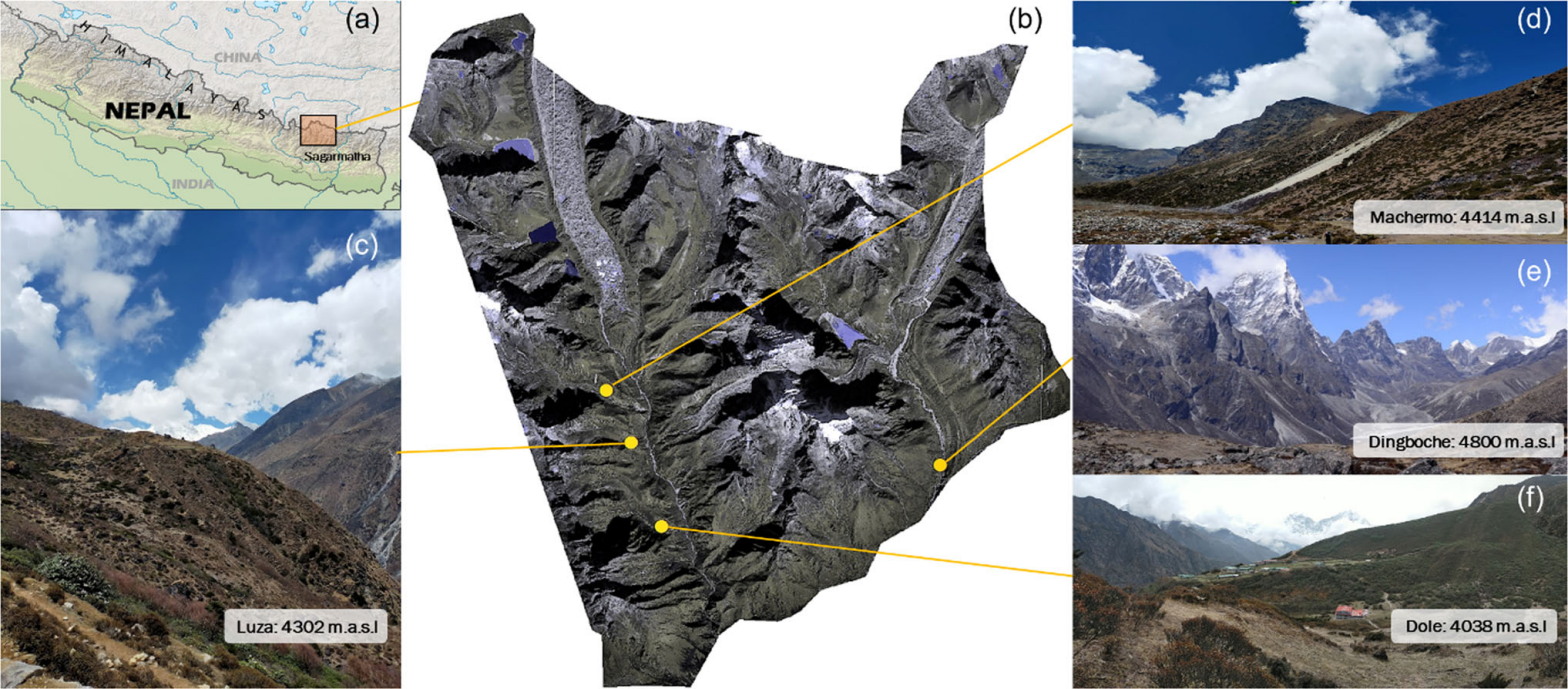
Illustrating typical alpine ecosystem in the Himalaya—These photographs depict four areas within Sagarmatha National Park, Nepal. **a** shows the location of Sagarmatha National Park; **b** shows imagery from WorldView2 of the key alpine region in Sagarmatha; **c**–**f** show four sites where grasses and dwarf shrubs are the dominant vegetation types, with sites located in Luza (4302 m.a.s.l), Dingboche (around 4800 m.a.s.l), Dole (4038 m.a.s.l) and Machermo (4414 m.a.s.l). Photographs are author’s own

In this paper, we define the Himalayan alpine zone as occurring between 4100 and 6000 m.a.s.l, which is the region where dwarf plants and snow will  interact. Figure 2 shows the location of Sagarmatha National Park in the Khumbu region of Nepal showing more detailed information about typical characteristics of the Himalayan alpine zone. Recent work using remote sensing analyses has shown that this high-altitude system has a spatial extent that is between 5 and 15 times the area covered by permanent snow and ice, and vegetation in this system is expanding (Dolezal et al. 2016; Anderson et al. 2020).

This review explores how expanding vegetation in the high mountain systems of the Himalaya might impact hydrological processes. Owing to the relative scarcity of studies in the Himalaya, in this review, we use insights on snow–vegetation interactions from studies in other global mountain systems to argue that ecohydrological processes within Himalayan systems cannot be overlooked in a warming climate. Considering the extensive nature of the Himalaya and the diversity of conditions across the mountain range, the situation and conditions in the well-instrumented Khumbu region of Nepal are the focus of this paper as an exemplar for broader Himalayan considerations.

## Himalayan ecology

**Fig. 3:**
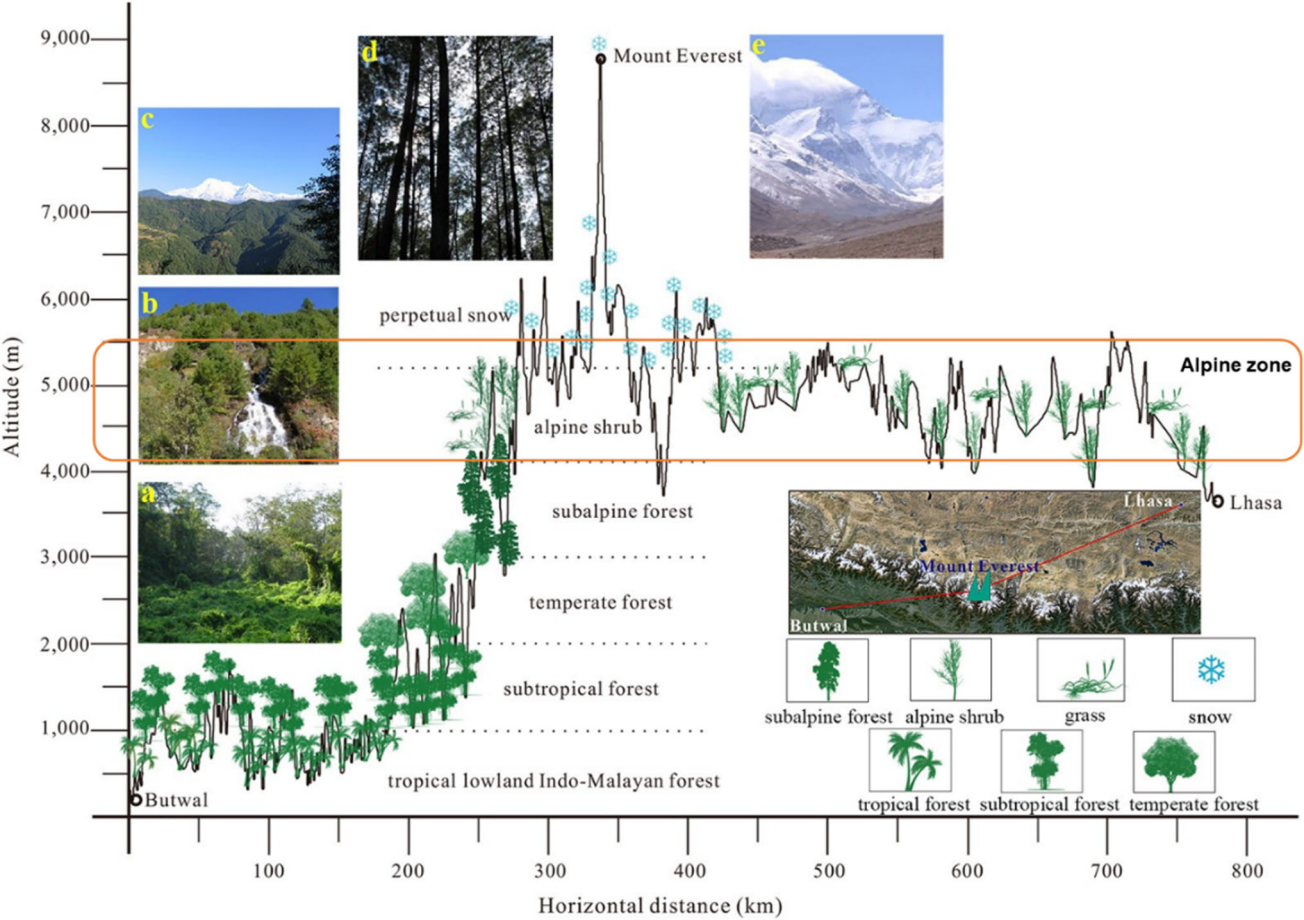
The vegetation distribution from Butwal in Nepal to Lhasa on the Qinghai–Tibetan Plateau. Photographs labelled **a**–**e** show tropical forest, subtropical forest, temperate forest, subalpine forest and perpetual snowy mountains, respectively. The alpine region considered in this paper is highlighted (Adapted from a figure in An et al. 2015, with permission)

Following the vegetation belts in Nepal defined by Joshi (1986), An et al. (2015) provided a sketch map of ecological zones and representative vegetation distribution from Butwal in Nepal to Lhasa on the Qinghai–Tibetan Plateau (Fig. 3).

**Fig. 4:**
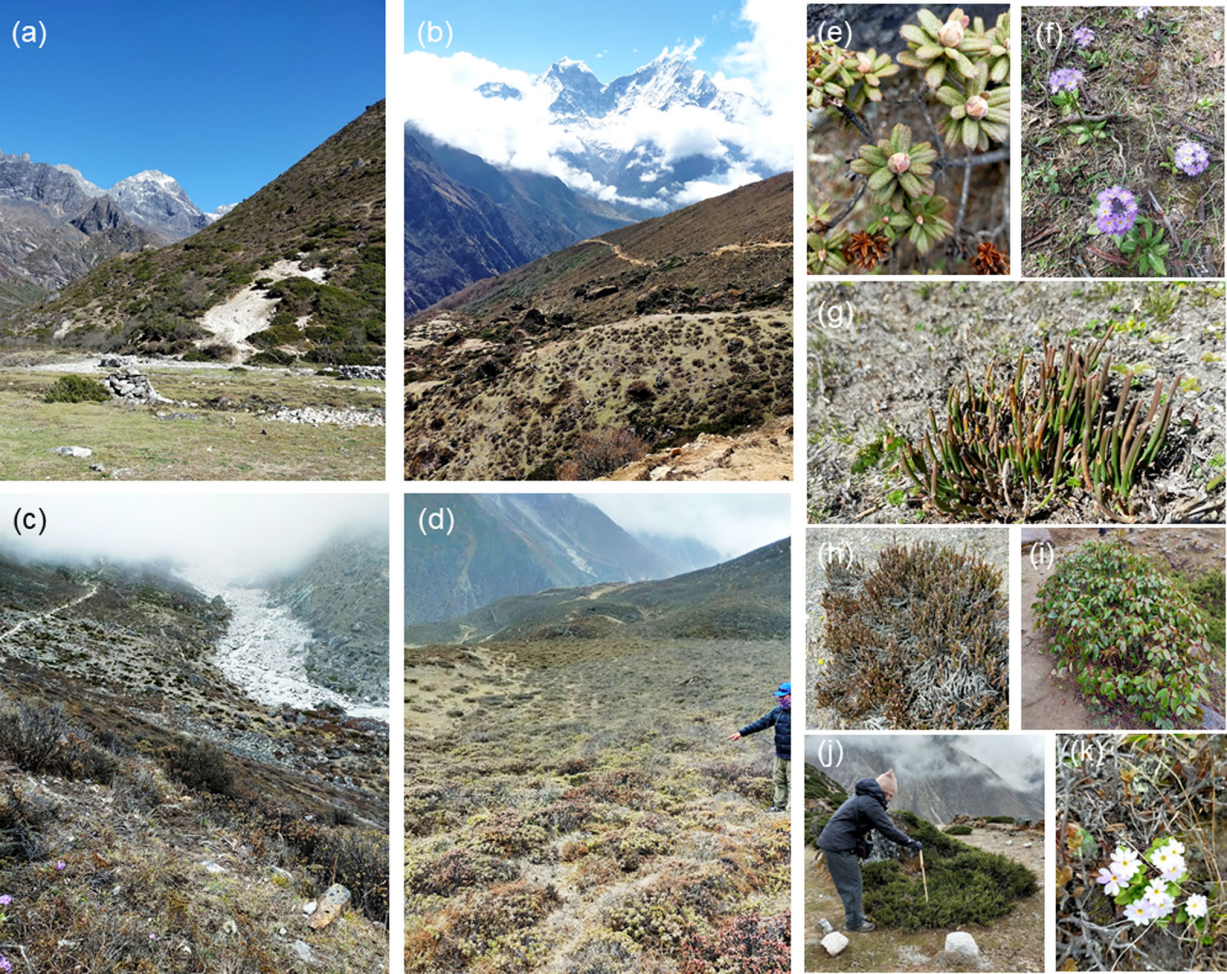
Landscapes, habitats and plants of the Himalayan alpine zone. These images were all captured by authors in the region between 4100 m.a.s.l. and 4500 m.a.s.l. in the Goyko valley of Nepal, within the Sagarmatha national park area of the Khumbu region. **a** is a mixed species hillslope near Dole (4100 m); **b** is an area between Dole and Machermo (~ 4300 m) comprising mixed *Juniperus indica* and various dwarf *Rhododendron* shrub species; **c** is a riparian zone above Machermo (~ 4500 m) comprising dwarf shrubs, predominantly *Rhododendron spp*; and **d** is a mixed area near Machermo (~ 4400 m) comprising dwarf *Rhododendron anthopogon* and *Rhododendron setosum* plants. In all cases, plant heights rarely exceeded 50 cm. Smaller photographs show common species within this zone—**e** *Rhododendron setosum* (typical height 30 cm); **f** *Primula denticulata* (typical height 10 cm); **g** *Ephedra gerardiana* (typical height 10 cm); **h** *Cassiope fastigiata* (typical height 10 cm); **i** *Rhododendron wallichii* (typical height varies considerably from 40 cm to 2 m); **j** *Juniperus indica* (typical height 40–60 cm); **k** *Primula walshii* (typical height 2 cm)

The lower lying temperate forest (2000~3000 m.a.s.l) transitions into subalpine forest between 3000 and 4100 m.a.s.l. The area between 4100 and 5000 m.a.s.l is named the ‘alpine’ zone where steppes, heath with dwarf plants and alpine meadow are the main communities (Golovatch and Martens 2018). Between 5000 and 5500 m.a.s.l is the ‘sub-nival’ belt, which is ecologically more barren, with lichens on exposed rocky surfaces and a few hardy flowering plants. Above this zone lies the region of permanent snowfields, rocks, glaciers and ice which (i.e. the ‘nival’ region) at elevations exceeding 5500 m.a.s.l (Ives and Messerli 1990). In this paper, we consider the whole area above the treeline and below the snowline and term this the ‘alpine zone’—encompassing the alpine, sub-nival and nival areas within which plant life could exist; we define this region as occupying the zone between ~ 4100 to approximately ~ 6000 m.a.s.l. We have set the upper limit of our consideration to 6000 m.a.s.l because this is approximately the highest elevation at which vascular plants have been found growing in the Himalayan region (Angel et al. 2016), although some geographically isolated field investigations have broadened the distribution of vascular plants in the Himalayas to 6150 m.a.s.l (Dolezal et al. 2016; Das et al. 2020) in isolated places. According to Dolezal et al. (2016), the root-zone temperature and the soil properties are the most important drivers for plant colonisation and growth, making exposure and micro-topography crucial for the establishment of plants. Hence, the establishment and migration of plants at the highest elevation regions on Earth have indivisible relationships with snow storage (affecting soil and land surface temperature) and snow melt (affecting the moisture and nutrient exchange with soil). Figure 4 shows some typical species within the alpine zone in the Sagarmatha national park area, as an exemplar of the stature and type of plants encountered above the treeline.

*In-situ* investigation of plant species distributions has been the predominant methodology for determining vegetation distribution along high-altitude gradients in the Himalayas, and owing to the remoteness of the region such surveys must typically be done on foot. Resultantly, there are limits to the extent of such data, limiting mapping to localities that can be accessed via paths or tracks, and these approaches are also restricted technically due to complex topographic conditions and the immense area of the Himalayas (Erinjery et al. 2018). Indeed, comprehensive herbaria and digital ‘collections’ of mountain flora exist, for example in the ‘Flora of Nepal’ catalogue at the Royal Botanic Gardens, Edinburgh^1^, and within Elizabeth Byer’s ‘Wildflowers of Mount Everest’ App^2^. However, whilst these collections provide detailed information about identification characteristics for individual species, there is scant information about the specific geographical distribution of those species. Accordingly, we argue that there is an urgent need for products that map also the spatial distribution of vegetation beyond accessible areas. Remote sensing provides one possible means of doing so, but requires detailed validation data to drive vegetation classifications and test accuracy of products. Multispectral satellite data have been shown to be useful for mapping and monitoring land cover in global mountain systems (Vaglio Laurin et al. 2013; Yu et al. 2017). However, the spatial complexity (heterogeneous vegetation mosaics often with small vegetation patches; Laurin et al. 2013), topographic-driven variability (Erinjery et al. 2018), and similarities of spectral information between key cover types and plants traits (e.g. shrub and grass), present challenges to applying this method to the Himalayan ecosystem above the treeline, and underline why validation using *in-situ* measurements is absolutely critical.

## Climate of Nepal

Understanding the spatial and temporal dynamics of climatic parameters (temperature, precipitation and snowfall) in the Himalayan systems is a priority for exploring the ecohydrological implications resulting from climate change. In this section, we outline the current status of meteorological monitoring in Himalayan areas, using Nepal as a case study.

**Fig. 5:**
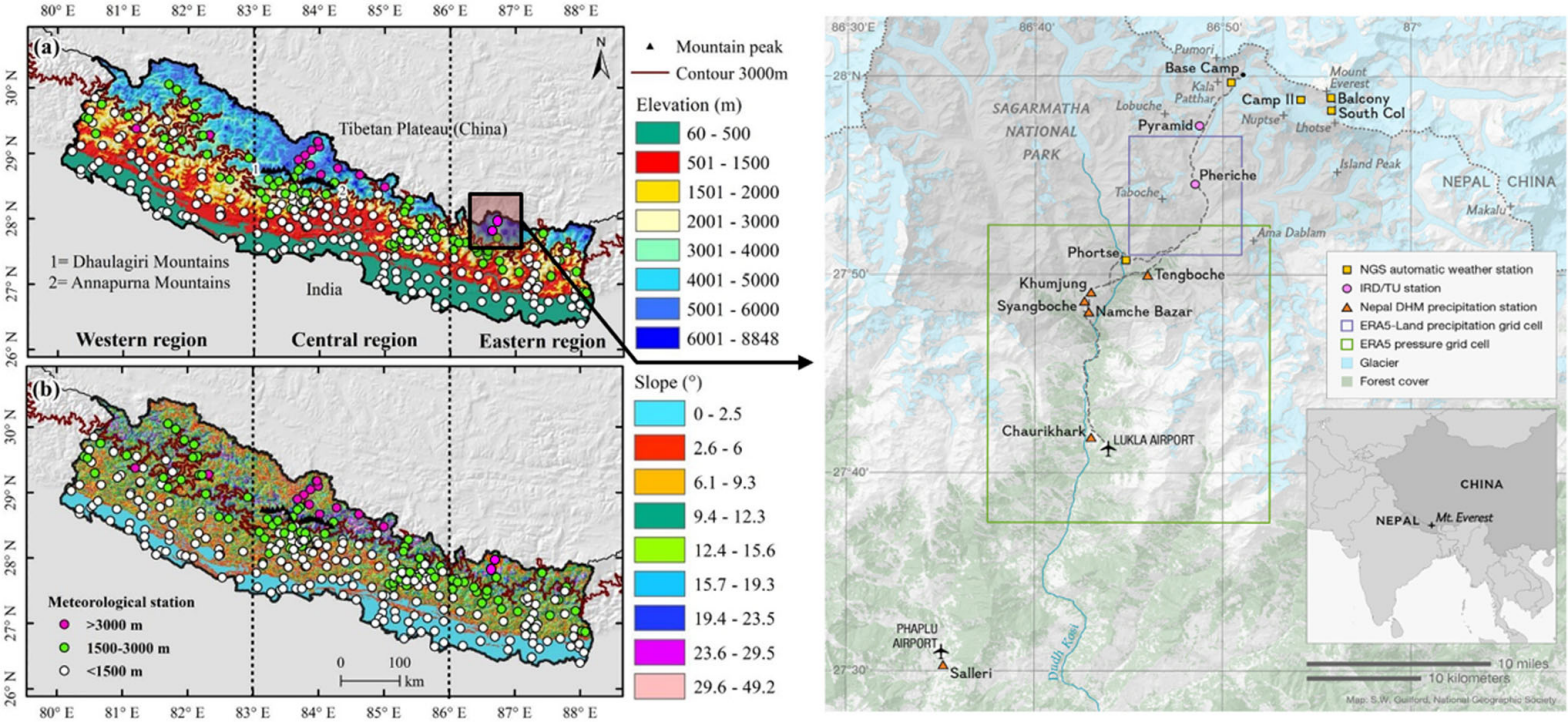
Left panel shows the location of all meteorological stations across Nepal with the digital elevation model as the background. White, green and cyan dots denote the stations located below 1500 m, between 1500 and 3000 m and above 3000 m, respectively. The black and black dotted lines are the national border and the subregion boundaries, respectively (Adapted from figures in Chen et al. 2021, with permission). Right panel shows weather station locations within the Sagarmatha national park area (Map by S.W. Guilford, National Geographic Society, with permission). Of those shown, the only stations within the alpine zone at altitudes above 4100 m.a.s.l. are Pheriche, Pyramid and Base Camp. Camp II, Balcony and South Col are above 6000 m.a.s.l. and therefore outside of the current bounds of the defined alpine zone

Surface gauge-based observations are the only direct method of obtaining long-term and high accuracy weather observations (Chen et al. 2021), especially in the Himalaya where abrupt changes in topography give rise to different climatic zones and weather patterns within a short latitudinal range (Chen et al. 2021). In Nepal specifically, the South Asian monsoon contributes 97% of the annual precipitation (Immerzeel et al. 2014; Mishra et al. 2014; Perry et al. 2020; Dahal et al. 2020; Hamal et al. 2020) . The climatological seasons in Nepal are defined by the monsoon period: the pre-monsoon season (March–May), summer monsoon season (June–September), post-monsoon season (October–November) and winter season (December-February; Nayava 1980). As the monsoon approaches from the East, the east and central areas receive the highest amount of monsoonal precipitation, with the lowest amount in western Nepal (Chen et al. 2021). As a result, the whole country can be divided into three district regions according to the differences in precipitation seasonality and patterns: the western (western boundary to 83°E), the central (from 83° to 86°E) and eastern region (from 86°E to eastern boundary; see black dotted lines in Fig. 5). Due to the influence of topography (i.e. the wet convective monsoon air mass being topographically forced), there can be a strong variation in climate parameters over short horizontal distances. The spatial distribution of meteorological stations varies considerably throughout Nepal, and ~ 90% of the stations are located below 3000 m.a.s.l (Fig. 5; Table 1; Chen et al. 2021), with only eight well-documented operational stations in the alpine zone above 4000 m.a.s.l (Singh and Mal 2014; Shea et al. 2015).

The difficulty of routine maintenance and remoteness complicates data collection at high altitudes (Dahal et al. 2020; Chen et al. 2021). Of the eight stations in Nepal above 4000 m.a.s.l depicted in Fig. 5 (left panel), three are located within the Khumbu area, near Mount Everest in eastern Nepal. These are Pheriche (4260 m.a.s.l), Pyramid (5035 m.a.s.l) and Everest Base Camp (5315 m.a.s.l), and they supply continuous weather measurements between 2016 and the present day (Table 1; Perry et al. 2020). In addition, there are four further high-altitude weather stations within Sagarmatha National Park which are not on the map in Fig. 5 (left panel), since these have only recently been installed, all of which also lie above the alpine zone at elevations exceeding 6000 m.a.s.l. (Table 1).

**Table 1:** Summary of weather stations in Khumbu, Nepal.  Data from Perry et al. (2020)

Weather station	Latitude (°N)	Longitude (°E)	Elevation (m.a.s.l)	Operation	Measurements
Salleri	27.5051	86.5862	2383	1 Jan 1948–31 Dec 2019	Daily precipitation
Chaurikharka	27.6965	86.7167	2642	1 Jan 1949–31 May 2020	
Namche Bazar	27.8022	86.7144	3450	1 Jan 1949–30 Sep 1983	
Syangboche	27.8114	86.7106	3700	1 Jun 1973–31 Dec 1979	
Khumjung	27.8189	86.7164	3750	5 May 1968–30 Dep 1991 1 Jan 1992–31 Dec 1992	
Tengboche	27.8333	86.7666	3875	1 May 1966–21 Aug 1979	
Phortse	27.8456	86.7472	3810	24 Apr 2019-present	Hourly precipitation and other meteorological variables
Pheriche	27.9090	86.8091	4260	27 May 2016–20 Nov 2019	
Pyramid	27.9583	86.8129	5035	27 May 2016–20 Nov 2019	
Base Camp	27.9952	86.8406	5315	10 Oct 2019-present	
Camp II	27.9810	86.9023	6464	9 Jun 2019-present	Hourly meteorological variables
South Col	27.9719	86.9295	7945	22 May 2019-present	
Balcony	27.9826	86.9292	8430	23 May-present	
Everest summit	27.9861	86.9226	8830	19 May 2022-present	

Understanding snow dynamics and subsequent influences on eco-hydrology requires quantification and measurement of regional energy balance variability. This is because low thermal conductivity and high albedo of snow insulates the land surface from incoming solar energy (Weller and Holmgren 1974), thereby strongly affecting climate processes and surface energy balance (Vavrus 2007). Driven by air temperature and precipitation, changes in snowfall pattern in high-altitude regions are reflected in changes in snow cover area (SCA), snow depth, shifts in snow accumulation and timing of snowmelt (Gurung et al. 2017). These changes impact downstream water balance since snow accumulation is the main contributor for water balance and peak runoff in mountain systems (Pomeroy et al. 2002). However, the sparsely located high-altitude weather stations in the region (Fig. 5) limit the availability of *in-situ* observations to improve understanding. Also, the recent climate records suggest that climate change has brought about increased variability in cryospheric snow distribution (Liston and Hiemstra 2011), which may become more significant in the alpine region as landscapes green in a warming climate (Keenan and Riely 2018).

From hereon, this review focuses on the dynamics of snow parameters (snow depth, snow cover dates, snow melt duration), and interactions with vegetation distribution (including vegetation composition and density) in high-altitude mountains. The review summarises the results from research in mountain systems globally, and seeks to summarise interactions between snow and vegetation—from these previous studies.

## Ecohydrological interactions in the alpine zone

### Plant influences on energy balance and temperature

Vegetation cover and biomass in alpine regions above the treeline are generally lower compared with tree-covered systems at lower elevations (Loranty and Goetz 2012). Over the past few decades, the cover of vegetation and the species diversity have been observed as expanding across many high-altitude mountain ecosystems globally (Okin et al. 2015; Anderson et al. 2020), and this includes the encroachment of shrub into former grasslands and tundra in the Arctic (Liston et al. 2002). Furthermore, Earth system models project that the world’s cold climate systems will green under climate change scenarios, as a function of temporal decline in temperature limitation for plant growth (Keenan and Riley, 2018). Vegetation expansion and transition in alpine regions alters the distribution of plant coverage and biomass, which impacts land surface energy balance (Huenneke et al. 2002). These drive climate processes (e.g. land surface temperature and humidity) and fundamental ecohydrological processes—including nutrient, heat and water cycles (Schlesinger 1990; McCarron and Knapp 2001; Fatichi et al. 2016).

Increasing grass density has been shown to alter soil heat fluxes owing to the different thermal properties between grass and bare soil surfaces; this leads to increases in soil temperature (D'Odorico et al. 2012), such that warmer land surfaces can alter the soil thermal conductivity (Romanovsky and Osterkamp 2000). This has been observed to trigger repeated freeze–thaw events which stress the microbial component, eventually causing microbe mortality (Schimel and Clein 1996). The substantial amount of nutrients released from microbial dieback during snow melt are critical to alleviate nutrient limitation for vegetation establishment in the next growing season, which is evident from studies in the Arctic (Schmidt and Lipson 2004; Li et al. 2020). It is hard to know whether the same processes would play out in the same way in Himalayan systems, so this warrants wider empirical investigation. As well as reducing radiative cooling, grassland with increased densities can modify other land surface attributes (including surface roughness, albedo and emissivity), which affects surface energy balance (D'Odorico et al. 2012). Work in the Tian Shan Mountains (Yang et al. 2020) has shown that low vegetation density reduces longwave radiation fluxes, leading to reduced near-surface temperatures—supplying the link between ecological and potential hydrological processes.

Compared with the transition from bare soil to grassland, shrub encroachment may bring about more significant influences on energy balance in mountain systems. Whilst these interactions have not been studied in Himalayan alpine systems, we can learn from studies elsewhere about potential interactions. For example, research in the Arctic is quite advanced in this regard and has shown that in snow-free periods, the structure of woody plants modifies the surface energy balance by decreasing albedo, increasing net radiation, reducing ground heat flux and increasing sensible heat flux to the atmosphere (McFadden et al. 1998). In winter, temperatures under shrub canopies that trap snow can be as much as 30 °C warmer than surrounding air temperature (Sturm et al. 2005), and these warmer temperatures can potentially enhance winter nitrogen cycling and lead to the release of larger pulses of nitrogen after snowmelt (Buckeridge and Grogan 2010). As a result, experiments in the Arctic show that snow–shrub interactions can create positive feedbacks to shrub growth and expansion by increasing nutrient availability in soils under shrub canopies (Sturm et al. 2005, 2001; Grogan and Jonasson 2006). However, the direction of influence from shrub encroachment on ground energy balance depends on soil types and local climate factors. In the Arctic permafrost thaw has been shown to be lower below tundra dwarf shrubs than in areas of wet sedge cover, indicating that soil below dwarf shrubs has a smaller heat flux (Juszak et al. 2016). It is therefore important to consider which effects might play out in Himalayan systems where shrub encroachment has been reported (Montané et al. 2007; Myers-Smith 2011; Formica et al. 2014), or if the co-impacts from meteorology, changed hydrology (e.g. soil wetness variations) and elevation conditions in the Himalayas might generate an unique impact on ecology balance caused by processes of vegetation change. More work is needed to investigate the scale and rate of processes such as shrub expansion and the potential impacts on hydrological processes in the Himalaya, and considering the particular plant traits of Himalayan species which may not exhibit precisely the same qualities as those reported in other cold climate regions of the world. Finally, it is likely that changes in plant cover or plant type may influence soil status, carbon content, aggregate stability and water retention but with a lack of studies in the alpine zone querying this, there is currently no information about how these factors might be influenced or changed, so new empirical work is urgently needed to address questions about plant–soil interactions, which could have wider impacts on microclimate and hydrology.

### Plant influences on snow storage

The physical structure of snow cover can insulate soil and air temperatures, which helps to maintain microbial activities and reduces energy losses from the land surface (Williams and Smith 1989). The predicted change of vegetation in the alpine zones of the Himalaya includes the expansion of vegetated area (i.e. increased plant density), and the transformation of vegetation composition (i.e. from herbaceous vegetation to woody plants). Given that the previous section has shown that plants can influence energy balance, it follows that they are also likely to impact snow storage.

The moisture exchange between soil and vegetation roots can enhance or decrease the amount of snow accumulation by changing the absorption of water from snowfall, snowmelt and precipitation (Walker et al. 1993). Hence, to a certain extent, the available moisture for vegetation establishment is determined by the type and density of plants. The higher vegetation cover translates to high root biomass under snow cover, which can increase water holding capacity in surface soils (McKinney and Cleland 2014). These impacts relieve the moisture limitation for plants growing in early spring (Li et al. 2020); thus more water from snow melt or summer rainfall can be retained in areas of dense vegetation cover than where vegetation is sparse. Also, compared with areas covered by bare soil and few plants, higher vegetation density can decrease land surface albedo in the snow season (Loranty and Goetz 2012). Working in the Arctic, Sturm et al. (2001) and Loranty and Goetz (2012) revealed that shrub expansion reduces albedo. Here, the land surface albedo in regions with shrub cover was 30% lower than under snow. This increased absorbed solar radiation by 69%–75%, increasing land surface runoff, and water supply for the subsequent growing season (Sturm et al. 2005; Marsh et al. 2010).

Shrub expansion can also influence snow storage owing to the comparatively taller canopy in shrubs compared to grasses and mosses (Wahren et al. 2005). In winter, shrubs can act like small, natural fences protecting snow from wind transport, hence an increase in shrub abundance may feedback positively on the distribution and persistence of snow under them (Sturm et al. 2001). The transformation from tundra to shrub composition in northeastern Canada increased snow depth by a factor of up to 3, whilst generating a decrease in snow density and cover (Busseau et al. 2017). With the exception of creating thicker snow storage under them, shrub canopies can also reduce the amount of moisture sublimated back to the atmosphere (Schmidt 1975; Liston et al. 2002). For instance, a regression model to describe snow and shrub cover showed that the increase of shrub cover in the Arctic increased the available water by 23%, which is consistent with the estimated loss of snow water equivalent (SWE) (by 10%–25%) in the wind-blown tundra areas (Sturm et al. 2001). Multi-year observations (1994–2001) of snow melt and vegetation distribution in Canada also showed that tall shrub snow accumulation was 147% greater than tundra (Pomeroy and Brun 2001).

Feedbacks from shrubland on snow properties have been observed in several mountain regions including Mediterranean Europe, USA, Australia and Argentina (Ludwig et al. 2007; Sandercock and Hooke 2011), although there appears to be little such research in the Himalaya. Work from other systems (e.g. drylands) has shown that plant-water interactions generated by the spatial distribution of vegetation, are small scale initially (i.e. at the scale of individual plants; Turnbull et al. 2012). Yet, they can exert large-scale effects because of the resulting changes to land surface runoff, soil moisture and resources together with the increased risk of soil erosion over large areas (Wainwright and Parsons 2002; Bartley et al. 2006; Okin et al. 2015). The uncertainties in our understanding of the interactions between vegetation composition and snow in global mountains complicate the description and understanding of how these might translate to the specific response of the Himalayan system. It is therefore an important and urgent research agenda to assess how plants interact with water flows and fluxes and how this might lead to disparate landscape evolution pathways, which could become further complicated due to temperature limitations and obvious impacts of elevation and aspect. Currently, the ecohydrological processes in the alpine zone receive no detailed consideration within major policy reports documenting future security for the Himalayan region (e.g. Nepal’s National Adaptation Plan Process 2016; The Hindu Kush Himalaya Assessment, 2019), and we assert that new data are needed to address this gap in understanding, and urgently.

### Plant influences on snowmelt

The process of snowmelt and the changes of snowmelt date influence vegetation growth, and induce strong ecosystem responses in several ways. Firstly, snowmelt timing controls soil moisture and nutrient availability in alpine regions, thus influencing plant growth rates and ecosystem functioning (Löffler 2007; Sutinen et al. 2009). An experiment to test the relationship between snow and soil microbial activities in mountain wetlands in the Alps showed that longer snow cover duration results in higher soil nutrient availability (Bombonato and Gerdol 2012). Secondly, snowmelt dates are associated with seasonal patterns of ecosystem carbon and water fluxes related to plant photosynthesis and growth (Galvagno et al. 2013; Rossini et al. 2014). A tight coupling of plant development with snowmelt dates was also observed in global high-elevation and high-latitude ecosystems (Wipf and Rixen 2010; Julitta et al. 2014; Vorkauf et al. 2021). Post-snowmelt temperatures were hypothesised to control plant phenology (Kudernatsch et al. 2008; Livensperger et al. 2016). Longer snow cover dates and larger snow cover fraction (SCF) lead to higher annual peak normalised difference vegetation index (NDVI) in Central Siberia and Kyrgyzstan (Grippa et al. 2005; Tomaszewska et al. 2020), which indicated that longer snow season and delayed snow melt were beneficial for soil accumulation which in term is linked to plant growth. Thirdly, snowmelt dates have impacts on the neighbourhood interactions between species (Wipf et al. 2006), e.g. between plants and herbivory, or pests and fungal species (Roy et al. 2004). Earlier snowmelt can be associated with an increased likelihood of damage from herbivory and fungi in the early growing season (Wheeler et al. 2016). These interactions may result in negative correlations between snowmelt dates and vegetation establishment, as highlighted by a study from the Italian Alps (Julitta et al. 2014).

### Plant influences on runoff

Vegetation can influence land surface runoff by altering infiltration, soil moisture budget and overland connectivity. Firstly, vegetation expansion usually increases root structures in the subsurface of soils, increasing soil cohesion, reducing or enhancing runoff and affecting soil infiltration characteristics (Abrahams et al. 1995; Osterkamp and Friedman 2000). The shrubbification of Arctic Alaska is linked to the increase of averaged snow depth by 14% (since the shrub-enhanced areas had deeper snow), thus leading to more surface runoff in the snowmelt period (Liston et al. 2002). Furthermore, thawing episodes triggered by increasing air temperatures have wide downstream consequences in mountain ecosystems (Trumbore et al. 1996; Maurer and Bowling 2014). Water tables in the active layer may decline with permafrost degradation (Walvoord and Kurylyk 2016), which changes regional hydrological regimes from predominantly surface flow to subsurface flow (Karlsson et al. 2016), thereby decreasing regional available runoff and ponding of water (Myers-Smith 2011; Connon et al. 2014).

Another impact of vegetation expansion is changes to the length of water transport pathways within vegetated areas, which has been demonstrated in drylands (Okin et al. 2015). High-density grassland systems with more continuous plant cover results in longer pathways for water transport (i.e. lower connectivity) than in more sparsely vegetated systems. Semi-arid studies have indicated that shrub establishment generates the same impacts on water transportation, which result in flashy increased runoff and decreased infiltration—a set of processes which further act to favour the growth of woody plants (i.e. a positive feedback mechanism for shrub encroachment; Schlesinger 1990; Okin et al. 2015). Whilst this work was conducted in semi-arid systems, similar principles of connectivity vs. dis-connectivity in hydrological processes probably apply to alpine zones with patchy (i.e. shrubs) vs. continuous (i.e. grassland) vegetation cover, although like in other areas highlighted by this paper, there is a lack of empirical data describing these processes in Himalayan settings, and so new research is needed.

### Uncertainties in snow–vegetation interactions

In mountains, marked altitudinal variation regulates atmospheric processes at a range of scales (orographic movement of wind, precipitation, condensation, wind drift, turbulent heat transfer, etc.), whereas the unique slope-aspect orientation controls land surface processes such as snow deposition, solar irradiation, surface energy balance and surface temperature (Sharma et al. 2014). These factors are important for governing the distribution responses within short-stature vegetation (Bennie et al. 2006; Körner 2021). Pape and Löffler (2016) demonstrated that the calorific energy of tundra and shrub in Norway vary with micro-topographic gradients instead of elevation, which verified that higher energy inputs from the sun and the resulting warmer microclimate are important factors for woody plant establishment and growth (Mesquita et al., 2018). Topographic drivers such as altitude and aspect also drive vegetation distribution patterns in mountain ecosystems, because they determine microclimate, insolation and the dissemination of seeds (Sharma et al. 2014). In addition, mass movements on steep slopes (Roe 2005; Roe and Baker 2006), disturb vegetation communities and contribute to differential patterns of meadow and shrub establishment. Hence, a high variability of phytomass and productivity along the micro-spatial topography results in higher ecological diversity only along the fine-scale gradients, where such micro-spatial diversity patterns are one of the main characteristics of the alpine environment (Pape and Löffler 2016).

Apart from the influences on vegetation establishment, these effects also result in heterogeneous distribution of snow across small spatial scales. A survey of snow depth in the U.S. Sierra Nevada showed that the snowpack in south-facing slopes receives higher solar radiation than those located on other slopes, accelerating snowmelt (Kirchner et al. 2014). Observations from weather stations in the western Himalaya showed that owing to the frequent avalanches and rapid melting, snow on the highest slopes exhibits maximum variability compared to other slopes (Misra et al. 2020). This small-scale pattern of varying snow depths results in highly variable ground surface temperatures at metre scales of up to 6 °C (Gubler et al. 2011; Gisnås et al. 2014). Influences and feedbacks between these factors and vegetation distribution are thus worthy of investigation, because increased avalanche risk might limit plant establishment, whilst changing soil temperatures linked to snow distribution might create patterns of more or less suitable areas for plant growth, with concomitant feedbacks to snow storage and melt over longer time periods.

## Key priorities for future research on Himalayan alpine eco-hydrology

**Fig. 6:**
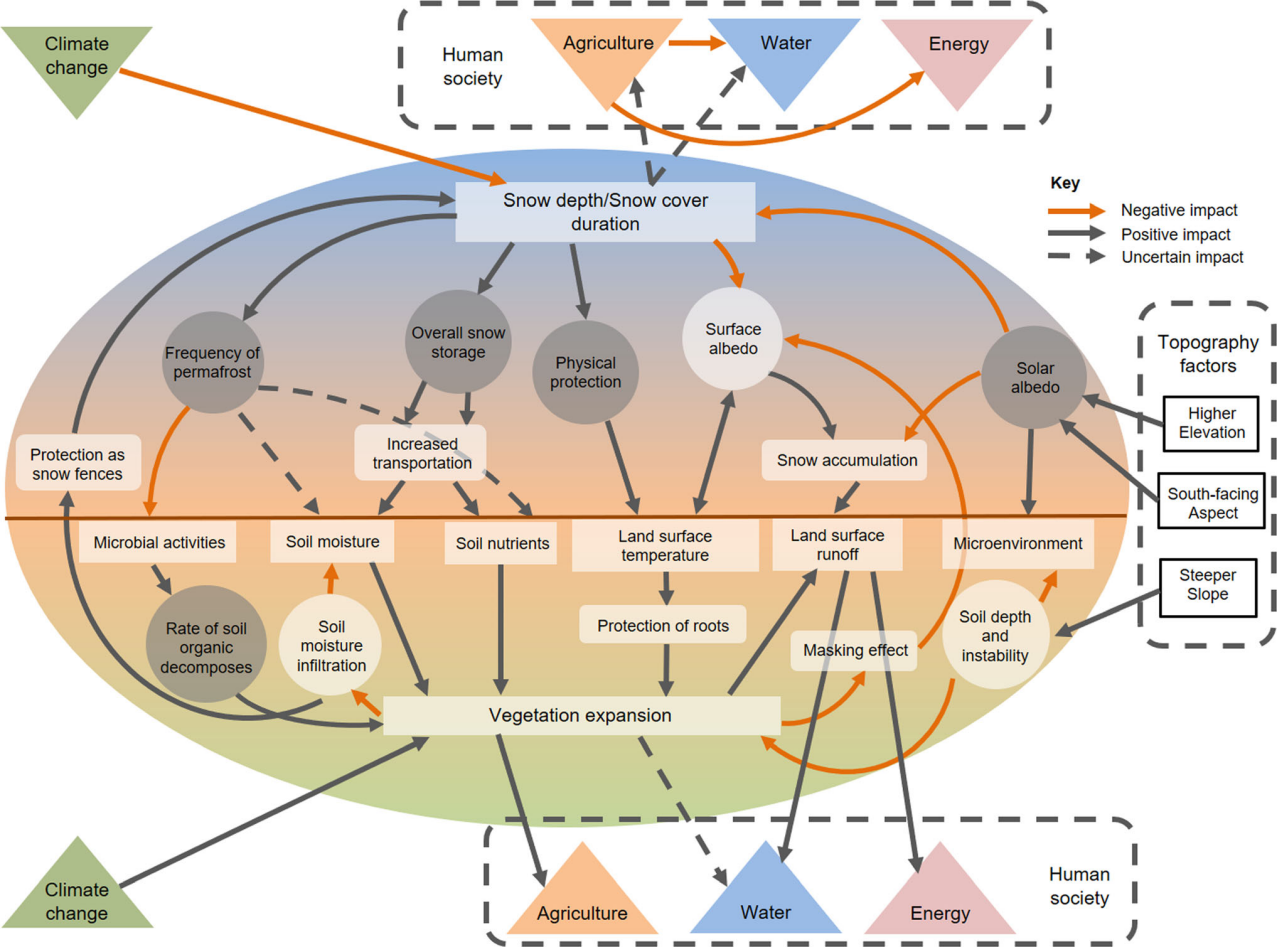
Impacts and feedbacks between hydrology, vegetation and soil processes in non-Himalayan systems and potential impacts on human society. There is insufficient evidence to be able to firmly say whether the strength and direction of these processes would translate from non-Himalayan to Himalayan systems so this remains a schematic for hypothesis testing and further investigation within the specific alpine zone of the Himalaya

We created Fig. 6 to consolidate the work reviewed in this piece, although readers are urged to exercise caution in translating directly the strength and direction of these feedbacks and relationships from non-Himalayan to Himalayan systems. Without robust empirical data from Himalayan systems there is no certainty that processes reported elsewhere will directly translate because plant traits, and the magnitude and frequency of landscape processes may differ. Nevertheless, this provides a visual guide showing the likely major interactions between plants and hydrology, and provides a springboard for future hypothesis testing in relation to ecohydrology within Himalayan alpine zones. Here, the various interaction effects and feedbacks can be seen, along with areas of uncertainty, indicate by dashed arrows. Negative impacts (orange arrows) are those where work (usually in other geographical regions) has shown that the interaction effect causes less snow storage or less vegetation establishment, whereas positive impacts (grey arrows) are those where the interaction effect results in increasing snow accumulation or vegetation expansion.

As we have discussed, there remain considerable gaps in the basic characterisation of alpine processes in the Himalaya, and a paucity of data to underpin evidence-based evaluation of past trends, current processes and future impacts of vegetation change at high altitude. In this section, we will discuss some of those gaps in our knowledge, and propose ways of building new understanding of processes, mechanistic interactions and future trajectories. Specifically, we focus on two areas where gaps could be filled by new work –

1. Understanding of *in-situ* ecological, hydrological and meteorological conditions in the Himalayan alpine zone above 4100 m
2. Understanding of alpine ecosystem snow and vegetation dynamics in the Himalaya

### In-situ biotic and abiotic conditions in the Himalayan alpine zone

As was discussed in “Himalayan ecology” and “Climate of Nepal” section, there is a paucity of ecological and meteorological information, which limits both site-based understanding of conditions and processes, and also prevents adequate robust validation of remote sensing retrieved data (see also “Spatial and temporal dynamics of plants and snow in the Himalayan alpine zone” section). Alongside, there is a need to consider site and regional variations in related factors such as edaphic, topographic and geological factors and how these interact with ecology and hydrology—but doing so is also hampered by the lack of data and poor instrumentation in high-altitude Himalayan regions. There have been some commendable efforts to instrument high-altitude catchments for monitoring e.g. seasonal mountain water cycles within glaciated areas (Steiner et al. 2021), and ecological experiments which have monitored transects in Himalayan systems at are increased in areas of steep topography (e.g. landslides and floods might threaten infrastructure)). Another layer of complexity is added because mountain regions are also characterised by “political and economic marginality” (Dolezal et al. 2016), and in comparison to surrounding lowland regions the people living at high altitude tend to have “little or no voice in national affairs” despite mountain regions being net exporters of resources (e.g. water) to populations living on the plains (Byers and Sainju 1994). . The lack of instrumentation at high altitude is therefore a function of poor infrastructure and lack of consistent electricity supplies, coupled with the operational challenges and high financial costs of installing and maintaining long-term equipment at altitude.  . Community involvement could take a minimalist form of wage labour, or it could be much better integrated if the communities can be given a voice in designing the monitoring program to address concerns they have themselves identified, such as water supply, natural hazards, quality of pasture vegetation for livestock or medicinal plant availability. So, there is a particularly pressing priority to try to improve the monitoring situation at high altitude, taking care to meaningfully involve and engage local citizens and represent local interests. Doing so would mean that impacts can be better understood and would ensure that local communities can benefit from the enhanced information about their locale, enabling any new data to drive improvements in management and conservation opportunities.

We argue that the shortage of high-altitude monitoring should be addressed through installation of more numerous monitoring stations for measurement of meteorological, hydrological and ecological processes. To an extent, such long-term monitoring requirements for ecology, hydrology s of these efforts, and such approaches could build on previous work which has reported on community led monitoring for e.g. air pollution in formerly poorly instrumented areas (Wong et al. 2018) and in remote Arctic communities (Johnson et al. 2015). Furthermore, there is evidence that involving residents in scientific experiments can help demystify science whilst also integrating community knowledge, and increasing uptake of the resulting data (Israel et al. 1998). From a scientific perspective this approach would also enable a means of community engagement which moves beyond ‘citizen science’ to formalised collaboration with citizen researchers, decolonising science and reducing the necessity for carbon intensive international flights to service equipment and download data. Sherpa communities in Sagarmatha National Park and other touristic areas of the Himalaya are now reasonably well connected via internet services although this is not consistent across all Himalayan areas, and may be poor away from major trekking routes. For basic weather stations and phenocams, however, power could be enabled e.g. through local governments or non-governmental organisations such as ICIMOD (International Centre for Integrated Mountain Development). Funding would be required to support community engagement, but if done sensitively, local communities could benefit from the increased scientific activities, increasing the attention directed towards otherwise marginalised communities overlooked by other funding^3^.

**Fig. 7:**
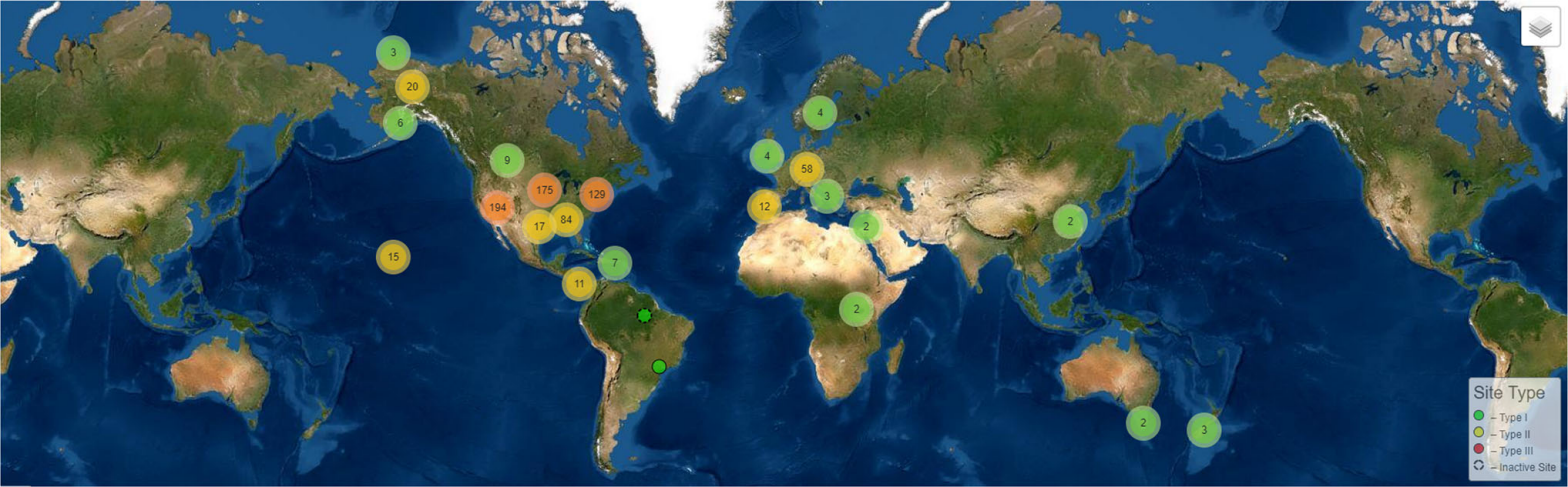
Current distribution (accurate February 2022) of phenocams globally showing a lack of sites in the Himalaya (see footnote 3)

**Fig. 8:**
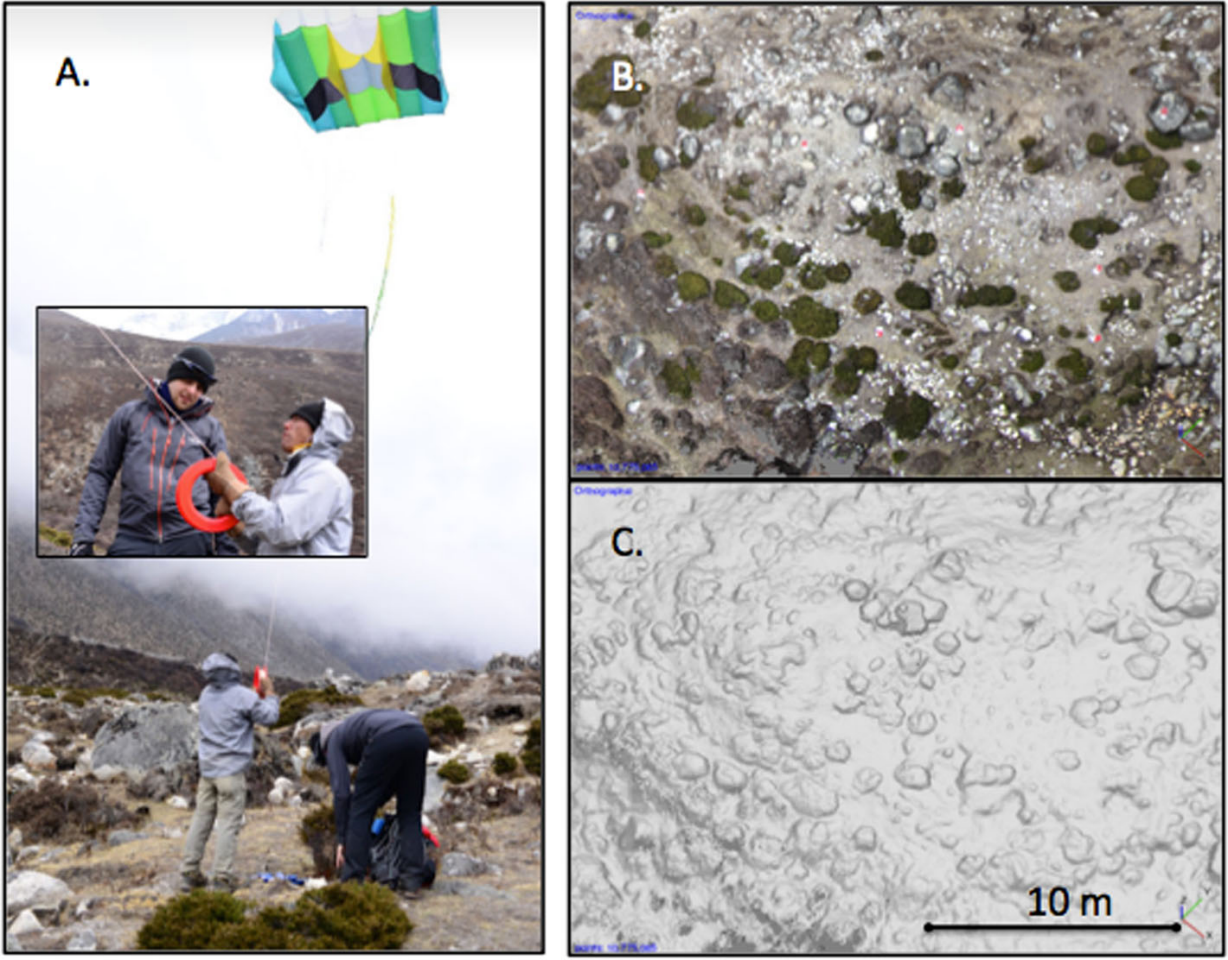
Results from kite-based capture of aerial data over shrub-dominated ecosystems found at around 4300 m in the Himalaya during fieldwork in 2017. Shown are **a** images of the kite aerial photography method, demonstrating (inset) the kite being flown by a skilled Sherpa operator; **b** the resultant orthomosaic derived from overlapping photographs captured using structure-from-motion photogrammetry processing, and **c** the spatial/structural information content of the structure-from-motion results for shrub canopy height measurement

In addition to monitoring data, at the local level there is also a lack of understanding about plant structures and properties and dynamics over time. New data are needed that describe the specific spectral properties of major alpine plants in the Himalayan region—since spectral properties will influence energy balance effects. Data from simple sensors such as phenocams (Brown et al. 2016) may go some way to improving understanding of seasonal dynamics and brightness variations, but currently there are no phenocams in the entire Himalayan region (Fig. 7). We suggest that installation of some high altitude phenocams (which can use simple and readily available technology such as digital repeat photography, timelapse cameras or adapted trail-cams (Sonnentag et al. 2012)) would provide a useful first step in understanding Himalayan phenology. Additionally, work that advances understanding of the structure of alpine plants is necessary because the structure of individuals and the spatial ecology of plant communities (particularly plant spacing and volumetric characteristics) will dictate hydrological processes such as snow trapping, shadow casting and thus snowmelt processes runoff patterns and hydrological connectivity. Owing to the relatively short stature of alpine shrubs, grasses and mosses, this calls for fine grained data. Whilst various options exist, in theory for this, the most pragmatic approach in the high-altitude alpine zone is to use a structure-from-motion photogrammetry method applied to kite-acquired aerial photographs (Fig. 8). Photogrammetric methods for processing overlapping aerial photographs are now relatively mature and allow both volumetric plant or plot characterisation (Cunliffe et al. 2016) and carbon estimation (providing allometric relationships for individual species can be determined (Cunliffe et al. 2021). The reason we do not propose drone-based monitoring above 4000 m is because researchers may face various problems with drone workflows at such elevations caused by low air mass, limiting vertical lift, which can limit endurance in multirotor platforms quite significantly. Whilst fixed wing systems may be less severely affected, drones themselves attract considerable attention in the Himalaya—and in many areas it is expensive to obtain research permits to use drones (a recent quotation for doing so in the Sagarmatha National Park was around $2000 for a single permit). Finally, there are practical limits—battery charging capabilities become more difficult as elevation increases and communities become more off grid and remote. For this reason, kite-based aerial surveys are optimally suited to such applications and data can be processed using the same workflows as used with drones (Duffy et al., 2018), and furthermore will allow (as discussed also in previous paragraph) enhanced engagement with local communities who can become involved in data acquisition which uses relatively simple ‘appropriate technology’.

### Spatial and temporal dynamics of plants and snow in the Himalayan alpine zone

The spatial and temporal variations of ecohydrological parameters in the Himalayan alpine zone are a core concern for local residents since they relate to water security and management, agriculture development and energy transformation (Paudel 2016; Nepal et al. 2021). For example, water availability for geoponic irrigation is essential for agriculture which is also a crucial export commerce in Nepal (Paudel 2016) and for the future potential of hydropower stations (Nepal et al. 2021). Although establishment of more numerous weather stations at high altitude would offer an improvement in site-based understanding of alpine processes there is considerable variation in parameters due to the monsoon climate (generating intra-annual variation; see  “Climate of Nepal” section) coupled with the extreme topography (leading to marked spatial heterogeneity; see 4.5; Nepal et al. 2021). Hence, satellite observations with fine resolution are necessary to complement these *in-situ* observations to harmonise monitoring of key variables.

Remote sensing methods provide an up-scaled perspective from which ecohydrological understanding can be derived—e.g. particularly through analysis of the spatial and temporal dynamics of alpine vegetation and seasonal snow. For example, there is potential for ecological information (e.g. land cover type, vegetation phenology, canopy cover and traits) derived from *in-situ* survey to be extrapolated to regional estimates via satellite-derived measurements, with an additional opportunity to hindcast using archived remote sensing data. During the monsoon season, cloud cover can mask the land cover signal in optical satellite imagery limiting high-quality seasonal/intra-annual observations of terrestrial parameters (Stendardi et al. 2019; Heckel et al. 2020). On the other hand, active remote sensing missions which use cloud-independent RADAR sensors offer an opportunity to overcome this limitation (Drush et al. 2012). Furthermore, we argue that the fusion of optical satellite synthetic aperture radar (SAR) data (twin satellites in Sentinel-1 (S1) mission) offer a pragmatic solution for the monitoring of high-altitude mountain environments (Drush et al. 2012; Heckel et al. 2020). In particular, such approaches have shown high accuracy for the prediction of meadow phenology (Stendardi et al. 2019), forest cover (Heckel et al. 2020) and crop classification (Chakhar et al. 2021). This approach also demonstrates the potential for mapping detailed land cover in alpine zones—for example, Arctic tundra height has been measured previously with C-band SAR (Bartsch et al. 2020), although validation data are still required for mapping dwarf plants in the Himalayan alpine zone. Apart from a great quantity of *in-situ* field work with apparently high expense of time and finance, validation could also be derived via commercial satellite data offering fine spatial resolution data (e.g. WorldView, QuickBird)—which offer optical and multispectral products at spatial resolution as fine as 0.46 m per pixel. An alternative participatory method for validation would be to exploit mountaineering photography (both contemporary and historic) since mountain expeditions to the world’s highest peaks have regularly been charted via photographic methods dating back to the early part of the twentieth century.

One limitation of extending the spatial and temporal scale of snow monitoring is the mismatch between the coarse pixel resolution of widely applied snow products and the high heterogeneity of land surface snow distribution in the Himalaya. For example, the most popular satellite-derived snow products come from the Moderate Resolution Imaging Spectroradiometer (MODIS) but these data have a spatial resolution of between 250 m and 1 km (Hall et al. 2002). Such resolution can severely restrict the high-quality retrievals of snow parameters in alpine Himalayan systems, because the change in topography that occurs at such scales is often very high, and this can lead to changes in e.g. solar radiation and corresponding nival processes (Roe 2005; Stillinger et al. 2019). Furthermore, clouds have similar reflective properties in visible and near infrared (NIR) bands with snow meaning that discrimination between snow and cloud from optical sensors is challenging (Stillinger et al. 2019). SAR data offer an attractive alternative for snow monitoring, with the cloud penetration properties of SAR reducing the limitation from cloud coverage and solar illumination (Dong et al. 2018; Lievens et al. 2019), demonstrating the capacity for snow estimations in mountain regions where the satellite-based estimates are currently lacking (Lievens et al. 2019). The fusion of Sentinel-1 (S1) and optical satellite data e.g. MODIS, Landsat-8 or Sentinel-2, provide comprehensive information and offer a possible solution to overcome the limited revisit frequency of S1 imagery—since the sparser S1 images (6-day revisit time) may not always capture accurately the corresponding snow conditions (Snapir et al. 2019; Tsai et al. 2019). S1-derived snow products such as snow cover extent, snow water equivalent and snow depth have been used effectively in Greenland (Buchelt et al. 2021) and the European Alps (Lievens et al. 2019) but not so far in Himalayan settings. Another inevitable limitation of S1-derived snow metric modelling is the limited length of the time series that can be generated, whilst S1 and convergence datasets may not capture the full historical variability of ecohydrological process behaviour, nevertheless this shortcoming will improve as more years of data become available (Snapir et al. 2019). The priority for future studies targeting snow in alpine zones should evaluate the transferability of advanced snow product algorithms from non-Himalayan regions to the Himalaya, and the convergence of high accuracy snow products derived from satellite missions with *in-situ* measurements mentioned in “In-situ biotic and abiotic conditions in the Himalayan alpine zone” section. The implementation strategies for future *in-situ* and spaceborne monitoring of key snow and vegetation parameters discussed in “In-situ biotic and abiotic conditions in the Himalayan alpine zone and “Spatial and temporal dynamics of plants and snow in the Himalayan alpine zone” sections are summarised in Table 2.

**Table 2:** Summary of the key parameters and implementation strategies for *in-situ* and spaceborne monitoring in the alpine Himalayas

Target parameters		Target parameters	Implementation strategies in Nepal	Reference(s)
*In-situ* monitoring of snow	Snow cover extent Snow cover date Snow depth Snowfall	Phenocams Laser monitoring station Automatic weather station with specific sensors	Time-lapse imagery (wildlife camera with specific mode) Soil pressure transducer	Bokhorst et al. (2016), Kirham et al. (2019)
*In-situ* monitoring of vegetation	Plant properties (species, height, canopy area) Plant distribution Vegetation phenology Spectral information	Quadrat/species survey of vegetation Structural model from photogrammetry Portable spectrometer	Kite-based aerial surveys	Duffy et al. (2018), Beamish et al. (2020), Zeb et al. (2021)
Spaceborne monitoring of snow	Snow cover extent (SCE) Snow depth Snow cover date Snow grain size Snow Water equivalent (SWE) Snow classification	Snow products derived from optical imagery (e.g. MODIS) SAR products (e.g. Sentinel-1, Radarsat, PALSAR, ASAR)	Data fusion of optical imagery with passive microwave data Improve ground validation to increase the reliability of satellite retrievals Snow–vegetation interactions should be explored	Dietz et al. (2012), Tsai et al. (2019)
Spaceborne monitoring of vegetation	Aboveground biomass Leaf Area Index (LAI) Vegetation phenology Net primary productivity Vegetation pigments Solar-induced chlorophyll fluorescence Classification and mapping Community composition, plant functional type, and fractional vegetation cover	Various optical satellite products: MODIS/AVHRR/Landsat/Sentinel-2 Very High Spatial Resolution (VHSR) commercial satellite imagery: WorldView/QuickBird	Exploit free-to-access data via platforms such as Google Earth Engine Snow–vegetation interactions should be explored	Beamish et al. (2020)

## Conclusion

This review demonstrates the importance of ecohydrological processes in the Himalayan alpine zone, whilst also underlining the current paucity of understanding about ecology, hydrology and meteorology conditions. We assert that ecohydrological functioning of water–plant interactions in the Himalayas will become a more significant concern under climate change as mountain systems ‘green’ (Keenan and Riley 2018; Shannon et al. 2019; Anderson et al. 2020; Körner 2021), and we suggest that these interactions will be most strongly felt in the alpine zone where multiple factors interact (e.g. physical (albedo changes); ecological (community composition, competition) and hydrological (storage, runoff processes); Fig. 1). Studies in high-altitude mountain systems, summarised here, have demonstrated that expanding dwarf plants above the treeline could deliver ecohydrological changes in addition to changes in water supplies driven by glacial mass balance loss. By highlighting ecohydrological influences and feedbacks learned from other systems, our review identifies the different and even opposing impacts arising from these processes, whilst also stating the environmental and geographical uncertainties. This context dependency emphasises the necessity of validating the extent to which findings from non-Himalayan regions translate to the Himalaya, and highlights that increasing the level of targeted monitoring efforts, even in small regional scales or short time scales will be valuable for advancing understanding in Himalayan alpine zones.

Although we recognise that conducting field investigations in the alpine Himalaya represents a huge challenge, we assert that this is an urgent research imperative since the subsequent processes have the potential to influence the water, energy or agricultural security in the wider region. We conclude that there is an urgent need for the establishment of new alpine zone monitoring stations, and community engagement with alpine science. Additionally, there is a need for synthesis of *in-situ* measurements and satellite-based parameters are priority solutions to plug current gaps in basic science that will advance understanding of ecohydrological processes in the Himalayan alpine zone. Implementation of these recommendations should ideally be considered by global organisations, since the alpine Himalayan water–plant interactions require greater understanding, environmental protection and increased social responsibility given the potential widespread consequences of anthropogenic-driven climate change.

## Footnotes

1 (http://www.floraofnepal.org/)

2 (https://stories.rbge.org.uk/archives/33162)

3 https://phenocam.sr.unh.edu/webcam/network/map/
